# Therapeutic vaccine for chronic diseases after the COVID-19 Era

**DOI:** 10.1038/s41440-021-00677-3

**Published:** 2021-06-08

**Authors:** Hironori Nakagami, Hiroki Hayashi, Munehisa Shimamura, Hiromi Rakugi, Ryuichi Morishita

**Affiliations:** 1 Department of Health Development and Medicine, Osaka University Graduate School of Medicine, Suita, Japan; 2 Department of Geriatric and General Medicine, Osaka University Graduate School of Medicine, Suita, Japan; 3 Department of Clinical Gene Therapy, Osaka University Graduate School of Medicine, Suita, Japan

**Keywords:** Antibody, Epitope, Vaccine

## Abstract

There is currently a respiratory disease outbreak caused by severe acute respiratory syndrome coronavirus 2 (SARS-CoV-2). After rapid development, RNA vaccines and adenoviral vector vaccines were approved within a year, which has demonstrated the strong impact of preventing infectious diseases using gene therapy technology. Furthermore, intensive immunological analysis has been performed to evaluate the efficiency and safety of these vaccines, potentially allowing for rapid progress in vaccine technology. After the coronavirus disease 2019 (COVID-19) era, the novel vaccine technology developed will expand to other vaccines. We have been developing vaccines for chronic diseases, such as hypertension, for >10 years. Regarding the development of vaccines against self-antigens (i.e., angiotensin II), the vaccine should efficiently induce a blocking antibody response against the self-antigen without activating cytotoxic T cells. Therefore, the epitope vaccine approach has been proposed to induce antibody production in response to a combination of a B cell epitope and exogenous T cell epitopes through major histocompatibility complex molecules. When these vaccines are established as therapeutic options for hypertension, their administration regimen, which might be a few times per year, will replace daily medication use. Thus, therapeutic vaccines for hypertension may be a novel option to control the progression of cerebrovascular diseases. Hopefully, the accumulation of immunological findings and vaccine technology advances due to COVID-19 will provide a novel concept for vaccines for chronic diseases.

## Introduction

The current respiratory disease pandemic coronavirus disease 2019 (COVID-19) is caused by severe acute respiratory syndrome coronavirus 2 (SARS-CoV-2). Although individuals with COVID-19 usually are asymptomatic or have mild symptoms, moderate and severe symptoms, including pneumonia, have been observed in some cases. RNA and adenoviral vector COVID-19 vaccines were rapidly developed and approved within 1 year. These types of vaccine technologies have been under development for cancer or genetic disorders in translational gene therapy research for >20 years; however, vaccines against SARS-CoV-2 have the strong impact of preventing infection through gene therapy technology [1]. Furthermore, immunological analysis (i.e., T cell epitope, T cell and B cell receptor repertoire genesis, and RNA-sequencing analyses) has been intensively applied to evaluate the efficiency and safety of vaccines, possibly allowing for rapid progress in vaccine technology.

We have attempted to develop novel therapeutic vaccines against hypertension, dyslipidemia, Alzheimer’s disease, cancer, and inflammatory diseases by targeting self-antigens. If the efficacy and safety of vaccines can achieve an effect equivalent to that of medication, vaccines may be an alternative to daily medication for the treatment of lifestyle diseases. Here, we describe our therapeutic vaccine concept and summarize the current knowledge on vaccines for hypertension.

## Overview of COVID-19 vaccines

**Fig. 1:**
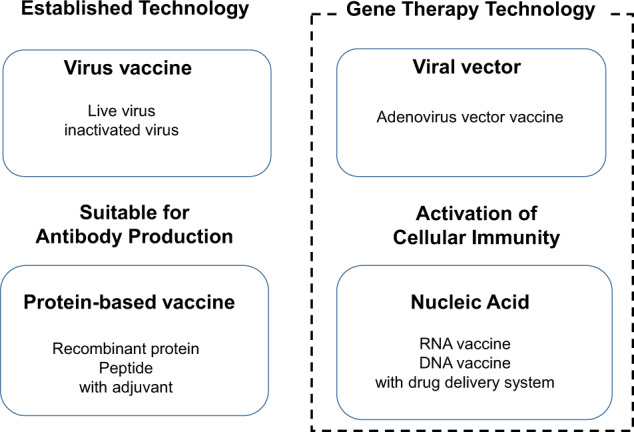
Four different types of vaccines. SARS-CoV-2 vaccines have been actively developed worldwide and are classified into four different types of vaccines (virus-based vaccine, protein-based vaccine, viral vector-based vaccine, and nucleic acid-based vaccine). Virus-based vaccines include inactivated and attenuated viruses, which are common vectors for preventive vaccines for infectious diseases. Protein-based vaccines include recombinant proteins, an outer shell that mimics viruses, such as virus-like particles (VLPs), or peptides, which are usually suitable for inducing antibody production. This vaccine type may require coadministration of adjuvants to stimulate innate immunity, leading to an efficient adaptive immune response. Viral vector- (i.e., adenoviral vector) and nucleic acid-based vaccines (i.e., RNA and DNA vaccines) utilize gene therapy technology. RNA vaccines and adenoviral vector vaccines have been rapidly approved worldwide

To combat the worldwide COVID-19 pandemic, the development of an effective and safe vaccine against SARS-CoV-2 was urgently needed. Researchers are developing vaccines against SARS-CoV-2, including adenovirus-, DNA- and RNA-based and inactivated vaccines [2–5]. SARS-CoV-2 vaccines are classified into four groups (virus, protein, viral vector, and nucleic acid) based on the technology applied (Fig. 1). Intact target viruses are well established and common as preventive vaccines for infectious diseases. Viral vectors are used to efficiently deliver genetic materials into cells, and adenoviruses have been commonly utilized as viral vectors. Vaccines involving DNA or RNA are included in the nucleic acid vaccine category.

In this last type of vaccine, DNA or RNA corresponding to a viral gene or modified gene is delivered into cells in the body to provoke an immune response. These vaccines can also be developed to use genetic material, not viruses, and potentially activate cellular immunity as well as humoral immunity. However, one of the problems for clinical development is the low transfection efficiency of nucleic acids in the body. In the rapid development of RNA vaccines against SARS-CoV-2, BioNTech and Moderna established gene delivery technology, which includes modification of nucleic acids and encapsulating mRNA in lipid nanoparticles. These companies rapidly, within half a year, presented initial results of clinical trials to support their RNA vaccine concept [6, 7], and in a phase 3 clinical trial, the RNA vaccine was found to be 95% effective in preventing COVID-19 [8]. The Food and Drug Administration (F.D.A.) granted the Pfizer and BioNTech vaccine the first approval given by the United States to a coronavirus vaccine, and the World Health Organization approved it for emergency use. Similarly, Moderna presented the initial results of a dose-escalation clinical trial [9, 10] and ultimately determined that its vaccine had an efficacy rate of 94%. The Moderna vaccine was the second one authorized by the F.D.A., at 1 week after approval of the Pfizer and BioNTech vaccine. Although these vaccines are highly effective in preventing COVID-19 in the short term, their safety and efficiency should be continuously monitored for a long time.

Among the four groups of vaccine technologies, the protein-based vaccine strategy, which includes the formulation of a recombinant protein or peptide, is known to induce antibody production, though this type of vaccine usually requires adjuvants that activate innate immunity. Importantly, the combination of a selected antigen and appropriate adjuvants may be a key molecular feature of therapeutic vaccines for chronic diseases. After the COVID-19 era, the novel vaccine technology developed will dominate among preventive vaccines for infectious diseases and therapeutic vaccines for chronic diseases.

## Mechanism of therapeutic vaccines against self-antigens

For many years, we have aimed at developing therapeutic vaccines for chronic diseases, such as hypertension. As described above for SARS-CoV-2 vaccines, the responses induced by vaccines usually mimic the immune response against pathogens and stimulate cellular immunity and antibody production through coactivation of innate immunity and antigen presentation by antigen-presenting cells (APCs). In most cases, the therapeutic vaccine target for chronic diseases is a self-antigen (i.e., angiotensin (Ang) II) and not a pathogen or virus. Therefore, we and others have developed therapeutic vaccine systems against self-antigens, which is somewhat different from preventive vaccine approaches for infectious diseases [11].

**Fig. 2:**
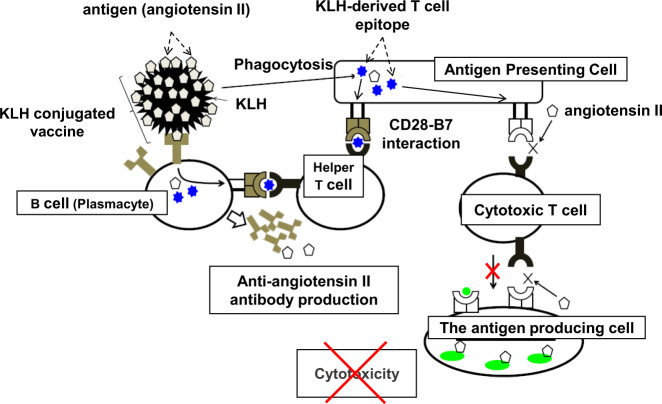
Scheme for KLH-conjugated vaccine against self-antigens. An antigen (i.e., angiotensin II) is conjugated with KLH and used in cotreatment with adjuvants. Antigen-presenting cells phagocytose antigen-carrier protein conjugates and present a T cell epitope of KLH to T cells on major histocompatibility complex molecules. Cotreatment with adjuvants effectively induces the CD28-B7 interaction through the activation of innate immunity. Thus, helper T cells recognize the corresponding epitope through T cell receptors, leading to proliferation and differentiation. With the help of activated T cells, B cells differentiate into plasmacytes and proliferate. Consequently, B cells produce antibodies targeting the antigen (i.e., anti-angiotensin II antibody). As the antigen (angiotensin II) does not include a T cell epitope, cytotoxic T cells are not activated by the target antigen and do not attack angiotensinogen-producing cells. KLH indicates keyhole limpet hemocyanin

First, humans possess an immune tolerance system to prevent autoimmune reactions to self-antigens, and this system can be disrupted to provoke the production of antibodies specific against self-antigens. The major mechanism of immune tolerance is T cell tolerance, which includes central and peripheral tolerance in the immune tolerance system. Central tolerance is known as “negative selection”, whereby T cells recognizing self-peptides displayed by major histocompatibility complex molecules (MHCs) are removed during their development in the thymus. After central tolerance, peripheral tolerance, known as “anergy”, is the second branch of immune tolerance. In particular, during interaction between T cells and APCs, the former cannot be activated without the CD28-B7 interaction, and cotreatment with “adjuvants” can contribute to activation of the CD28-B7 pathway through innate immunity to overcome T cell tolerance. To activate B cells to produce antibodies specific for self-antigens, CD4-positive helper T cells are required for B cell differentiation into plasma and memory cells. Thus, with antigen and adjuvant cotreatment, self-reactive B cells function with the stimulation of CD4-positive cells [12] (Fig. 2).

Second, selective activation of humoral immunity has been proposed by a therapeutic vaccine for a self-antigen. Preventive vaccines usually activate both cytotoxic T cells (cellular immunity) and antibody production (humoral immunity) to eliminate the pathogen or virus; in contrast, therapeutic vaccines for chronic diseases should efficiently induce a blocking antibody response without provoking a cytotoxic T cell response. For this purpose, therapeutic vaccines require helper T cell activation for antibody production by B cells but not cytotoxic T cell activation. Therefore, as we cannot include T cell epitopes as antigens, peptide antigens are commonly used in combination with foreign T cell epitopes as carrier proteins. For example, Ang II (8 amino acids) has never been displayed on MHC class I or II in mice and humans, but an anti-Ang II antibody can be produced because B cells that produce such antibodies are found. In addition, follicular helper T cells (Tfhs) were recently shown to mainly contribute to efficient and sustained antibody production by B cells [13]. Tfhs, which are characterized by C-X-C chemokine receptor type (CXCR) 5, programmed cell death protein (PD)-1, inducible costimulator (ICOS) or interleukin (IL)-21 expression driven by Bcl-6, migrate to germinal centers in lymph nodes and interact with B cells. Moreover, Tfh-activated B cells can produce a given antibody with high affinity for a long period of time. Interestingly, the neutralizing antibody levels of COVID-19 patients correlate well with the number of peripheral Tfhs, which also supports the connection between Tfhs and antibody production [14]. Therefore, activation of Tfhs will induce antibody production, which may be the best approach for therapeutic vaccines for chronic diseases.

**Fig. 3:**
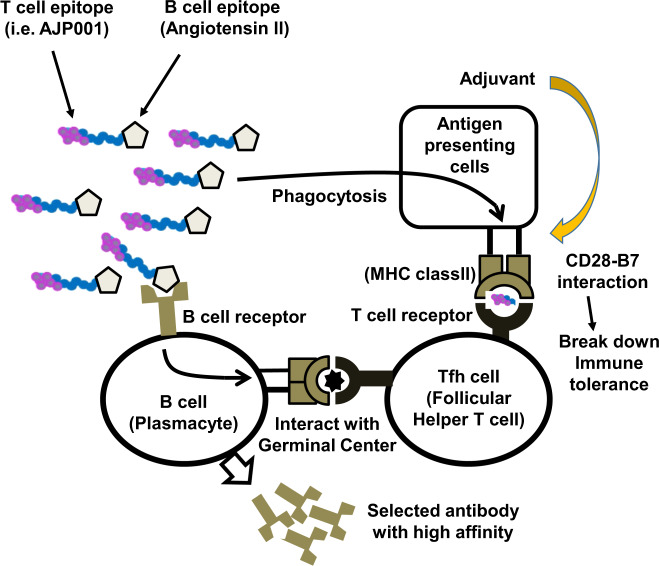
Scheme for a peptide epitope vaccine against self-antigens. A peptide epitope vaccine consists of an antigen (i.e., angiotensin II) with a conjugated T cell epitope (i.e., AJP001) and is coadministered with adjuvants. Antigen-presenting cells phagocytose the peptide vaccine and present a T cell epitope on major histocompatibility complex (MHC) molecules. B cells also take up the peptide vaccine via the B cell receptor and present a T cell epitope on MHC molecules. Cotreatment with adjuvants effectively induces the CD28-B7 interaction through the activation of innate immunity, which can overcome immune tolerance. Follicular helper T cells activate B cells to differentiate into plasmacytes and proliferate in the germinal center, contributing to sustained high-affinity antibody production by B cells. MHC indicates major histocompatibility complex

We modified a therapeutic vaccine for Ang II (self-antigen) based on the immune system. We have utilized keyhole limpet hemocyanin (KLH), a standard carrier protein that is immunogenic and contains a strong T cell epitope, and Ang II conjugated to glutaraldehyde [15] (Fig. 2); instead of KLH, we simply utilized a helper T cell epitope (named AJP001) and directly conjugated it to Ang II [16, 17]. After the first immunization (priming) with AJP001 and an Ang II peptide (AJP-AngII vaccine), APCs phagocytose AJP-AngII and present the helper T cell epitope AJP001 to T cells through MHC class II. Of note, it is important to avoid the presentation of Ang II itself through MHC. T cells then recognize the antigen through the T cell epitope and are activated within the context of innate immunity coactivation by adjuvants. Following the second immunization (booster), B cells phagocytose AJP-AngII via the B cell receptor specific for Ang II and present the helper T cell epitope, AJP001, to T cells through MHC class II. Thus, the combination of AJP-AngII and adjuvants induces the proliferation and differentiation of T cells specific for AJP-Ang II. Next, B cells differentiate into plasmacytes and produce antibodies with the assistance of T cells (Fig. 3). We confirmed T cell activation by AJP001 but not Ang II in T cell proliferation and enzyme-linked immunospot assays. Importantly, we selected short antigenic peptides (Ang II; 8 amino acids), without including a T cell epitope. Because MHC class I and II epitopes usually consist of 8–10 and 12–20 amino acids, respectively, short peptides with fewer than 8 residues are preferred as antigens from a safety point of view. Consequently, antibodies targeting Ang II were produced successfully and safely. In the next generation of therapeutic vaccines, we may propose an epitope vaccine including a combination of a helper T cell epitope and B cell epitope.

## Therapeutic vaccines for hypertension

Several preclinical and clinical trials investigating vaccines for hypertension have been reported in the last 20 years. We briefly summarize the therapeutic vaccine for each target in hypertension.

### Renin or prorenin

The first investigation reported the efficacy of vaccines targeting renin [18–20]. Although renin vaccines contributed to a reduction in blood pressure (BP) in animal models, immunological renal disease characterized by the presence of immunoglobulin and macrophage infiltration colocalizing with renin was observed at autopsy, which might be a severe risk of renin vaccines. Nevertheless, Qiu et al. recently reported the efficacy of a KLH-conjugated renin vaccine for reducing high BP without an autoimmune response [21]. Importantly, they designed a target peptide comprising only 7–10 amino acids, which is shorter than that used in the original vaccines and does not include T cell epitopes. As mentioned above, it is important to inhibit activation of cytotoxic T cells, which may cause discrepancies in autoimmune responses. We also designed a prorenin vaccine that does not cross-react with renin. Since prorenin contributes to the progression of diabetic retinopathy and nephropathy, independent of Ang II, a therapeutic vaccine targeting prorenin will be useful for the treatment of these diseases.

### Ang I or II

An Ang I vaccine conjugated with KLH was shown to reduce BP in animal models [22, 23]. However, the vaccine did not reduce BP in a randomized double-blind placebo-controlled clinical study, even though the anti-Ang I antibody titer increased [22, 24]. Ambühl et al. designed an Ang II vaccine (named AngQb-Cyt006) conjugated to virus-like particles (VLPs) [25] derived from the coat protein of the bacteriophage Qb, which acted as a carrier protein. The systolic BP of spontaneously hypertensive rats (SHRs) was significantly lower with than without vaccination, and the lowered systolic BP was equivalent to that of rats treated with ramipril. Similarly, we evaluated the efficacy of an Ang II-KLH vaccine [26]. Following Ang II infusion, systolic BP was significantly lower in immunized mice than in unimmunized mice. We also designed a DNA vaccine against Ang II [27], which resulted in not only BP reduction but also organ-protective effects, and Ang II-induced perivascular fibrosis in the heart was significantly attenuated in immunized mice and rats [26, 27]. Moreover, an Ang II vaccine was effective in preventing heart failure in a rat model of myocardial infarction with permanent left anterior descending artery ligation [28]. Wakayama et al. reported the effect of preexposure to Ang II vaccination on cerebroprotection after middle cerebral artery occlusion in rats [29], and the production of anti-Ang II antibodies was related to a reduction in infarct volume with suppression of Ang II type 1 receptor (AT1R) mRNA.

Several clinical trials of vaccines against Ang II have already been reported. Twelve healthy volunteers were injected with AngQb-Cyt006 (100 µg) in a placebo-controlled randomized phase I trial [25], and the anti-Ang II antibody titer increased in all subjects. Subsequently, a double-blind, randomized, placebo-controlled phase II a trial was conducted to investigate the effect of AngQb-Cyt006 injected three times at weeks 0, 4, and 12 in 72 patients with mild-to-moderate hypertension [30]. In the high-dose (300 µg) group (*n* = 24), the mean ambulatory daytime systolic BP was significantly lower than that in the placebo group (*n* = 24) at week 14. Furthermore, the high vaccine dose reduced early morning BP. Nonetheless, there was no significant difference between the low-dose (100 µg) and placebo groups. In terms of safety, none of the five serious adverse events were deemed to be treatment related, which indicates no safety concerns in this study. We also conducted a double-blind, randomized, placebo-controlled phase I/II a trial using an Ang II DNA vaccine (AGMG0201) to evaluate the safety, tolerability, pharmacokinetic, and pharmacodynamic effects, and exploratory efficacy of AGMG0201 administered to patients with moderate essential hypertension at two dose levels. This trial is ongoing and will hopefully provide important information regarding the development of therapeutic vaccines.

### AT1R (Angiotensin II type 1 receptor)

The efficacy of a therapeutic vaccine against AT1R has also been investigated. Chen et al. designed a peptide (named AT1R-001) derived from AT1R conjugated with VLPs [31]. AT1R-001 is derived from the second extracellular loop of AT1R, with the sequence C-A-F-H-Y-E-S-Q, a ligand-binding site [32]. BP was significantly decreased in immunized SHRs compared with unimmunized rats. Interestingly, anti-AT1R-001 antibodies and Ang II did not competitively bind to AT1R, whereas Ang II receptor blockers (ARBs) exhibited competitive inhibition of AT1R [33, 34]. Azegami et al. also reported that AT1R vaccination prevented NG-nitro-L-arginine methyl ester-induced nephropathy [35]. Therefore, antibodies specific for the AT1R second extracellular loop may have organ-protective effects, similar to ARBs.

### Calcium channel

Wu et al. reported the development of vaccines with VLPs against the L-type calcium channel for the treatment of hypertension in SHRs [36]. A peptide (named CE12) was derived from the epitope of the third extracellular region of domain IV of the human L-type calcium channel [37, 38], and after four injections, systolic BP was significantly decreased in vaccinated SHRs compared with unvaccinated rats. A bivalent vaccine against CE12 and AT1R also decreased BP in SHRs as well as glomerular injury induced by NG-nitro-L-arginine methyl ester.

## Future prospects

**Fig. 4:**
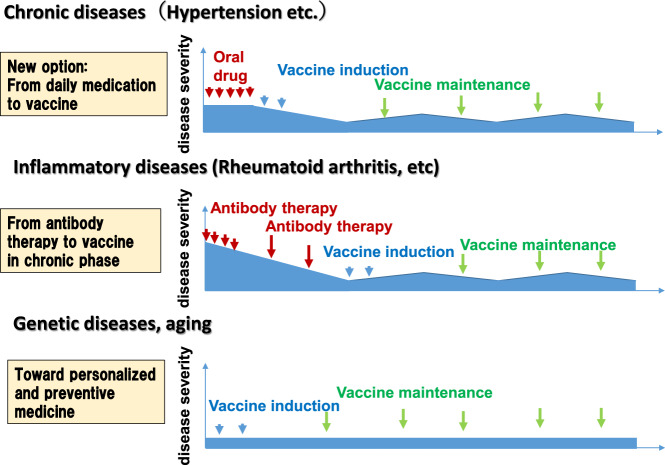
Future prospective of our therapeutic vaccine. First, some chronic diseases are lifestyle-related diseases, such as hypertension, and vaccination is being proposed as a new option for these diseases as an alternative to daily medication. Second, for inflammatory diseases (i.e., rheumatoid arthritis), switching therapy from antibody therapy to vaccination in the chronic phase is proposed because vaccines can possess a long window of action. Third, we will address the challenge of developing personalized and preventive vaccines for genetic diseases and aging in the future. The right panel shows disease severity in the acute and chronic phases, which can be regulated by vaccination in addition to oral drug or antibody therapy

Therapeutic vaccines for chronic diseases are moving toward clinical application. Here, we describe the future prospects of our therapeutic vaccine (Fig. 4). First, we aim to propose a new therapeutic option, namely, vaccination, as an alternative to daily medication for lifestyle-related diseases, such as hypertension. If a vaccine can partially replace daily medication for hypertension, diabetes, or dyslipidemia, this optional therapy would be able to contribute to improvements in drug adherence and polypharmacy in our aging society.

Second, for inflammatory diseases (i.e., rheumatoid arthritis) and cancer, several antibody therapies are effective in attenuating disease activity but are continuously administered for a long period. Thus, we propose a switch from antibody therapy to vaccination in the chronic phase but not the acute phase. Because a vaccine may possess a long window of action, this optional therapy should improve a patient’s quality of life and decrease medical costs. Third, we will address the challenge of developing personalized and preventive vaccines for genetic diseases and aging in the future. Although an antiaging vaccine is still a conceptual target in animal studies, we recently reported a therapeutic vaccine for senescence-associated T cells as antiaging senolytic therapy [39, 40].

Although there are many issues to be solved prior to clinical application, we believe that therapeutic vaccines will contribute to improving our health in the future.
